# Allergic sensitization: food- and protein-related factors

**DOI:** 10.1186/2045-7022-4-11

**Published:** 2014-04-15

**Authors:** Scott McClain, Christal Bowman, Montserrat Fernández-Rivas, Gregory S Ladics, Ronald van Ree

**Affiliations:** 1 Syngenta Crop Protection, LLC, 3054 E. Cornwallis Road, Research Triangle Park, NC, USA; 2 Bayer CropScience LP, 2 T.W. Alexander Drive, Durham, NC, USA; 3 Hospital Clinico San Carlos, IdlSSC, Servicio de Alergia, 28040 Madrid, Spain; 4 Agricultural Biotechnology, DuPont Experimental Station, Route 141 and Henry Clay Road, Wilmington, DE, USA; 5 Departments of Experimental Immunology and Otorhinolaryngology, Academic Medical Center, University of Amsterdam, Amsterdam, 1066GN, The Netherlands

**Keywords:** Allergic sensitization, Protein, Food allergy, Endogenous allergen, Food matrix

## Abstract

Presented here are emerging capabilities to precisely measure endogenous allergens in soybean and maize, consideration of food matrices on allergens, and proteolytic activity of allergens. Also examined are observations of global allergy surveys and the prevalence of food allergy across different locales. Allergenic potential is considered in the context of how allergens can be characterized for their biochemical features and the potential for proteins to initiate a specific immune response. Some of the limitations in performing allergen characterization studies are examined. A combination of physical traits of proteins, the molecular interaction between cells and proteins in the human body, and the uniqueness of human culture play a role in understanding and eventually predicting protein allergy potential. The impact of measuring food allergens on determining safety for novel food crops and existing allergenic foods was highlighted with the conclusion that measuring content without the context of clinically relevant thresholds adds little value to safety. These data and findings were presented at a 2012 international symposium in Prague organized by the Protein Allergenicity Technical Committee of the International Life Sciences Institute’s Health and Environmental Sciences Institute.

## Introduction

In April 2012, an international symposium titled “Sensitizing Properties of Proteins” was held in Prague, Czech Republic, bringing together over 70 scientists from academia, government, and industry. The purpose of the symposium, organized by the Protein Allergenicity Technical Committee (PATC) of the International Life Sciences Institute’s (ILSI) Health and Environmental Sciences Institute (HESI), was to present data on the current state of the science regarding the sensitizing properties of proteins in relation to food allergy [1-3]. Food- and protein-related factors are the focus of this paper.

Assessing sensitization potential is difficult to address because defining the characterization parameters that could be applied in a “safety testing platform” for food allergy risk have not yet been completed. Summarized here are some of the biophysical features of protein allergens, how and why allergen content is measured, and the way in which allergens interact with the immune system at the molecular level as well as at the human population level. Topics include 1) prevalence of known food allergens from a global perspective, 2) understanding endogenous protein allergenicity in the context of new foods and particularly genetically modified (GM) food crops, 3) food matrix effects on allergenic potential of proteins, and 4) proteolytic effects of allergens on sensitization potential.

### Prevalence of allergies around the globe

Tracking the prevalence of food allergy is considered critical to understanding how different populations become sensitized to allergens and where clinically relevant allergy emerges and persists. Characterizing geographically distinct allergens can help clarify the overall allergy disease process, which will someday help provide a path to predicting when, where and how allergy develops in individuals as well as populations. Understanding exposure and sensitization in distinct populations therefore has touchstones in characterizing not only known allergens, but charactering novel food proteins whether they arise from newly introduced foods or novel biotechnology. Although a mechanistic approach to understanding specific protein allergen behavior at the cellular level is necessary, prevalence is a bellwether that can alert scientists to regional or global changes. More than 150 foods have been implicated in allergic reactions, but the majority are induced by a small number of foods. In 1995, a Food and Agriculture Organization (FAO) technical consultation identified the following eight food groups as the most common causes of allergy worldwide: milk, egg, peanut, tree nuts, wheat, soy, fish and shellfish. These foods are known since then as the “the big eight allergens”, and are recognized as allergenic foods of public health importance. Therefore, they are included in regulatory allergen lists worldwide. Recent studies on prevalence are helping to support a broader inclusion of foods on the list of prominent allergenic foods, particularly in Europe, and some countries survey newly imported foods for allergenic potential. However, the 1995 FAO list of eight remains a foundation of how food allergens are prioritized.

The main criterion for inclusion in the big eight allergen list was the frequency of reported reactions. There are, however, some drawbacks to worldwide application of this list. First, epidemiological studies in food allergy in 1995 were limited, and even today, more than 85% of them have been performed in Europe, the United States (US), Canada, Australia and New Zealand. There are a few surveys carried out in Asia, and there is very scarce information about food allergy in Africa and Latin America. Second, recent meta-analysis and large scale reviews of food allergy epidemiology [4,5] have raised major issues about the comparability of studies due to marked heterogeneity in the design, the instruments applied, and selection of outcome measurements. Depending on the “definition” of food allergy, the overall estimate of prevalence changes with a progressive reduction: self-reported reactions > IgE sensitization > combination of symptoms and IgE sensitization > oral food challenges.

Milk and egg allergies are the most prevalent worldwide in children below four years of age. The prevalence based on food challenge studies has varied from 0% to 3% for milk, and from 0% to 9% for egg. Because tolerance develops spontaneously in the majority of patients (more commonly to milk), the prevalence is reduced in schoolchildren and adolescents, and milk and egg allergies reported in Australia are very uncommon in adults [4,6,7].

Peanut allergy is highly prevalent in the US, Canada, Australia and the United Kingdom (UK). It starts early in childhood with figures of 2.9% in one-year-old infants from Australia, affects around 1.5% of school age children and adolescents, and occurs in 0.7% of adults [4,6,7]. A lower incidence has been observed in Asia and Israel. In Japan, peanut induces only 2% of all food allergic reactions [8]. A survey carried out in schoolchildren in Singapore and the Philippines found a prevalence of peanut allergy of 0.43% to 0.67% among children of Asian origin, whereas in (western born) expatriate children it was 1.2% [9]. Similarly, the prevalence of peanut allergy in Jewish children in Israel was 0.17%, whereas that of Jewish children in the UK was 1.85% [4,7]. Interestingly, the prevalence of milk allergy in Israel was also lower, *i.e.*, 0.5% in children 3–5 years of age, which contrasts with figures of 1.8%-2.9% in children of the same age in the US and UK [4,7].

The prevalence of tree nut allergy as confirmed by oral challenge has ranged from 0.1% (almond in the UK) to 4.3% (hazelnut in adolescents 15–17 years of age in Germany) [5]. The prevalence in the US and Canada using a random telephone survey was 0.5% and 1% in adults, and 1.1% and 1.6% in children, respectively [7]. Similar to observations recorded for peanut, the prevalence of tree nut allergy in Asian children from Singapore and the Philippines was 0.3%, whereas in expatriate children it was 1.2% [9].

According to European studies, the prevalence of allergies to wheat and soy that included identification by oral challenges was 0%-0.5% and 0%-0.7%, respectively. However, serum-specific IgE to wheat was found in 3.6% of adults and to soy in 2.1%-2.9% of adults [5]. This discrepancy may reflect the presence of cross-reactive IgE antibodies with grass and birch pollen in patients without clinically relevant food allergy to soy or wheat being the main culprit.

In contrast to Western countries, wheat allergy is very prevalent in Japan, inducing 10% of all immediate reactions to foods, and it was ranked third for inducing allergic reactions after egg (29%) and milk (23%). It is also noteworthy that buckwheat (a non-cereal grain used in soba noodles) accounted for 6% of all food allergic reactions in Japan in one study, although the frequency doubled in patients older than 7 years [8].

The prevalence of fish allergy in studies carried out in Scandinavia, the UK, the US, and Canada varied between 0.2% to 0.6% [4,7]. In Japan, 5% of food allergic reactions were due to fish ingestion [8], demonstrating a substantial difference in prevalence compared to these other countries. The prevalence of shellfish allergy in studies performed in Europe and the US that combine symptoms and IgE sensitization was 0.6% in a study by Rona et al. [4]. In random telephone surveys performed in the US and Canada, the prevalence of shellfish allergy in adults was 2.5% and 1.7%, respectively, whereas in Canadian children the estimate was 0.5% [7]. In Asian countries, the overall prevalence of shellfish allergy was higher, and it is the most important food allergy in school age children, adolescents and adults [8,9]. In Singapore and the Philippines, the prevalence of shellfish allergy in schoolchildren of Asian origin was 1.2% in children 4–6 years of age and rose to 5.2% in those 14–16 years of age. In contrast, the prevalence for the same age groups in western-born expatriates was 0.55% and 0.96%, respectively [9].

Allergy to fruits and vegetables has been primarily investigated in Europe with scarce information available from other geographic areas. In Europe, the prevalence of fruit allergy confirmed by oral food challenges varied from 0.1% to 4.3%. The fruits most commonly involved are those belonging to the Rosaceae family (*e.g.*, apple, peach, and cherry) [5,10]. Allergy to vegetables is less frequent with prevalence in the general population occurring at 0.1% to 1.8% [5,10]. The vegetables most commonly involved in allergic reactions are those from the Apiaceae family (celery and carrot) and tomato [5,10]. One of the main features of allergy to fruits and vegetables is its frequent association with pollen allergies (pollen-food syndrome). The allergy progression begins with a primary sensitization induced by pollen exposure and the plant allergy appears later as a result of cross-reactive IgE to allergens found in pollens and foods. Examples include the Bet v 1 homologues and profilins. Allergies to tree nuts and peanut can also be linked to pollen allergy. For this reason plant food allergies are more frequently found in pollen allergic patients. In a study performed in Denmark, the prevalence of plant food allergies in (birch) pollen allergic patients was 19.2% for hazelnut, 16.7% for apple, 13.3% for kiwi, 7.6% for celery, and 5% for tomato [10].

Recently, within the EuroPrevall project (publication pending), two epidemiological surveys were carried out in schoolchildren and adults from the general population. In addition, more than 2000 patients from 12 allergy clinics across Europe were fully evaluated. All of these studies confirmed the importance of plant food allergies in schoolchildren and adults. The most prevalent food groups were (in decreasing order) fruits, tree nuts, vegetables, and peanut. Egg and milk were the most prevalent foods in small children; fish and shellfish were not common; and wheat and soybean allergies were very rare (unpublished data).

In summary, the most prevalent food allergies vary worldwide. As discussed, there can be dramatic differences regionally in the prevalence to a common food allergen. With priorities on important allergens being set by prevalence, different regions would be expected to have differing “top ten” lists of allergenic foods. There are important age and geographical differences in the prevalence of allergy to individual foods that depend on different dietary habits, environmental factors such as pollen exposure, and possibly genetics. More research is needed to fully delineate the global prevalence of food allergies, but it is expected that the EuroPrevall project will help shed light on this variable global phenomenon once all of the data are interpreted and disseminated.

### Variability of endogenous protein allergen levels in non-GM crops and their relevance to the safety assessment of GM crops

Speakers and participants engaged in a discussion about the relevance and purpose of quantifying endogenous protein allergen content in GM crops. The safety value of measuring the content of endogenous protein allergens in various food crops, particularly soybean, arose as a regulatory concern for GM crops in the last five years. Recent guidance from the European Food Safety Authority contains the precipitating language that ties endogenous allergen considerations to putative unintended GM effects: “with alterations to the allergenicity of the whole plant and derived products e.g. due to over-expression of natural endogenous allergens as an unintended effect of the genetic modification” [11]. The questions then are: What is the best way to analytically measure potential increases in allergen content? Can quantitative measurement of allergen content address allergenicity risk? The concern over GM crops is about the potential change in allergen levels due to gene manipulation and this was addressed at the symposium in the context of newly developed methods for measuring allergens.

Presumably, individuals with food allergies could minimize their symptoms by selecting food varieties with low allergen content. However, this is not a practical solution because foods are not assessed or labeled for their quantitative allergen content; rather, if assessed at all, foods are evaluated only for the putative presence of allergens. From a patient safety perspective, allergic individuals will typically avoid offending foods (whether GM or non-GM derived), thus reducing exposure to any level of endogenous allergen to a negligible level for any single variety (*e.g.*, registered GM variety) of a commodity crop such as soybean. If a conventional food crop is known to be allergenic (*e.g.*, soybean) and is already regulated as such (*i.e.*, via labeling), the question then can be asked: How important is a change in allergen content? In contrast to GM varieties, new non-GM soybean varieties can be and are introduced into the food supply with no requirement for a pre-market safety assessment and no required information on whether allergen levels have been altered. If this were a pertinent question unique to GM crops, then understanding natural variability of *in vivo* allergen content (in seed/grain) would be important. However, if the recognized inherent variability in allergen content [12] is not considered a safety issue for nontransgenic crop varieties, there is no evidence to suggest that a safety concern exists for GM varieties.

Currently, a robust safety assessment is in place for GM foods, and is performed according to regulatory recommendations; a full and thorough accounting of food and feed safety is maintained throughout the product’s life cycle and includes a proactive approach to stewardship. As part of the food safety risk assessment, some regulatory authorities require an assessment of endogenous allergen levels in certain GM crops (*e.g.*, soybean) which relies on a comparison to their non-GM counterpart. This drives towards a definitive yes or no answer as to whether changes have occurred in endogenous allergen levels despite a lack of knowledge regarding natural variability in the concentrations of specific allergens. Quantifying the amount of endogenous allergens in GM crops is of negligible value in a safety assessment of a particular crop variety where natural variability is not well understood, clinical threshold values of exposure are not established, and the variety itself is always mixed with other commodity varieties on the open market.

Across non-GM crop cultivars, endogenous allergen levels have been found to vary considerably due to genotype, environmental conditions, harvest timing, and/or storage conditions. For example, Ariyarathna et al. [13] reported up to a 15-fold difference in lipid transfer protein (LTP) levels in non-GM maize hybrids grown in Nebraska. In addition, most maize hybrids showed an increased amount of LTP in an un-irrigated location compared to two irrigated locations. Similarly, Kuppannan et al. [14] quantitatively evaluated LTP levels in 14 commercially available non-GM maize varieties using liquid chromatography (LC)-ultraviolet/mass spectrometry (MS) and reported LTP levels to vary between 58 and 678 μg/g (approximately 12-fold). Allergen levels in 20 varieties of commercially available non-GM soybean were found to vary up to 10-fold by quantifying multiple proteins by LC-MS [12]. Stevenson et al. [15] evaluated the influence of genotype and environment on allergen and anti-nutritional proteins in soybean using LC-MS and found that, for most allergens, the effects of environment were more important than differences between varieties brought about by breeding. The content of Gly m 4, the Bet v 1 homologue protein found in soybean, was measured in four non-GM varieties grown at five different locations in North America, and concentrations (μg/g of protein) were found to vary from approximately 2–7 fold. Thus, there appears to be marked variability in the levels of allergenic proteins in non-GM crops already in the food supply based on variety, abiotic and biotic stresses, and growing location. Interestingly, a recent literature review found that transgenesis had less impact on genome expression and concentrations of proteins or metabolites compared with conventional breeding or plant non-directed mutagenesis [16], highlighting the difficulty in hypothesizing that there is undue risk in increased protein allergen levels due to transgenesis.

It is currently not possible to correlate individual protein allergen concentrations with the risk of eliciting a clinically relevant response in sensitized individuals (*i.e.*, allergy risk) relative to the exposure to the protein through food because of the limited availability of data on quantitative thresholds for sensitized individuals. This applies to most allergenic foods which include the important commodity crops such as soybean. Presumably, future work would focus on using recent advancements in measuring individual allergens to better understand elicited patient responses in controlled exposure trials. Understanding individual allergen dose responses would be critical to establishing threshold exposure levels; yet this is just one aspect of the complicated nature of allergen exposure as it pertains to considering how allergens enter the human body in the context of the constituent food components in which protein allergens are embedded. This greater level of consideration for allergen exposure is discussed in the “matrix effects on allergenicity” section.

An important consideration for GM safety testing is to ask the question most pertinent for working towards an answer that can inform safety. Simplistically, the question may appear to be, What is the hypothesis that will support the comparison of the GM versus the non-GM crop? The premise behind this question appears to be that greater exposure to a protein(s) by consuming potentially higher concentrations in foods results in a higher frequency of sensitization within a population. This hypothesis, however, is not so straightforward. Greater exposure at a young age to allergens such as peanut, hen’s egg, or cow’s milk can have the opposite result [17,18], whereby increased exposure supports tolerance. There also remains a lack of clinically-based recommendations for safe consumption levels of allergenic foods and the processed consumables made from these foods. In addition, any discussion regarding how to address endogenous allergen content should observe the fact that the quantity of food consumed by a given individual is not subject to regulation; without this recognition, there is a limit to the ability to characterize exposure variability based merely on the concentration of an allergen in a food and still provide context for GM safety testing of endogenous allergens.

In summary, measuring precise levels of endogenous allergens is not warranted for GM food safety assessment purposes for the following reasons:

1) There is a lack of clarity on whether safety of GM crops is enhanced beyond existing guidance and actions that support safe GM products in the marketplace.

2) Interpretation of small concentration differences for allergens is problematic because information on exposure levels and sensitization thresholds is lacking for individual allergens. Crops such as soybean contain many individual allergen proteins.

3) Potential adverse effects cannot be directly deduced from measuring individual allergens (particularly in complex foods with multiple allergens), irrespective of improvements in the sensitivity of analytical detection.

4) It is impractical to distinguish between GM and conventional crop varieties in terms of clinically relevant endogenous allergenicity.

Until exposure thresholds to individual allergens are identified, there is no defined degree of added value to the safety testing of GM crops or any other allergenic food by quantitatively measuring allergens.

### Matrix effects on allergenicity

Foods consist of a complex mixture of substances collectively known as a food matrix. The matrix becomes an important factor in understanding sensitization to specific protein allergens because the allergen is never in initial contact with the immune system in a purified state; the matrix surrounds, interacts with, and can affect the physiochemical features of the allergens. Matrix components constitute the milieu of proteins, carbohydrates, phytochemicals, etc., from various parts of a plant or organism which may interact with an allergen of interest. Although a list of putative matrix components in plants alone is virtually endless, the study of food allergens has mainly centered around interactions with components in most abundance in the most commonly consumed foods. Matrices can be particularly important during the processing of foods where proteins can be exposed to pH changes, heat, and other environmental changes. Multiple guidance and consensus documents for assessing potential allergenicity cite the need to consider the effects of the food matrix. However, information regarding the role of the matrix in allergenicity is scarce. In contrast to analytically measuring allergens, there are no straightforward approaches to addressing the many variables represented by the matrix components in foods. As discussed, soybean is the primary focus of GM endogenous allergen considerations; yet there are no prescriptive approaches to quantitatively consider the impact of matrices, if indeed endogenous allergen hazards were identified. In this context, this is largely a discussion on the evolving science of the bioavailability of food allergens to the immune system.

Currently, the primary influences of the matrix on allergenicity are thought to be antigen bioavailability and release, digestibility, and interactions with the immune system. The matrix surrounding a protein of interest may contain other proteins, fats, carbohydrates, and numerous non-nutritive components with unknown potential for influencing allergenicity. Lipids can influence sensitization and the severity of elicitation responses via altered antigen release, digestion or immunomodulation. Matrix sugar content can alter digestion and impact the modification of allergens during processing, thus enhancing or diminishing allergenicity. Protein-protein interactions may also impact allergen digestion, and matrix proteins have the potential to directly stimulate the immune system. Additionally, most foods contain low levels of non-nutritive substances and contaminants, some of which may be immunomodulatory.

Antigen bioavailability and digestibility are closely linked and affect the quantity of intact antigen available for processing and presentation within the immune system. Digestibility, assessed *in vitro* and *in vivo,* is likely to vary depending on protein form (purified vs. present in a matrix). Particular food matrix structures, such as those in oilseeds, can delay the release of antigen after ingestion. Likewise, plant cell walls may not be broken down in the upper digestive tract. Although some matrix components have been shown to enhance digestion, most tend to inhibit digestion by restricting access of proteases to target proteins, outright inhibition of proteases, or competing for digestive enzymes (in the case of protein-rich matrices). The fatty acid phosphatidylcholine, a surfactant produced by the stomach and found in milk and egg yolks, has been shown to delay or inhibit *in vitro* digestion of milk allergens. Several studies demonstrate that pectin and other indigestible polysaccharides can inhibit digestion via reduction of pepsin activity, protein–pectin complex formation and viscosity [19].

Uptake and transport of proteins can be significantly affected by the presence of a food matrix. For example, hazelnut extract impedes the transepithelial transport of milk, hazelnut, and apple allergens *in vitro* and alters the kinetics of antigen transit into the blood of fed rats [20]. These effects are most likely due to the presence of non-allergen proteins competing for active transport machinery, given that the hazelnut extract was defatted. However, matrix lipids may influence antigen trafficking as well. Long-chain fatty acids have been shown to enhance the uptake of antigen into the plasma and mesenteric lymph nodes in mice and promote greater proliferation of antigen-specific T cells. In humans, a reduction in fat content can profoundly alter the clinical reaction to allergen. In one study, a double-blind placebo-controlled food challenge of peanut allergic patients was used to show that an approximately 9% reduction in fat in the peanut preparation reduced the amount of peanut required to elicit a reaction [21]. RAST-inhibition curves were not altered by the fat content of the mixtures, indicating that antibody-binding properties were unchanged. Thus, it is likely that a higher fat content served to slow allergen release and absorption. Lipids may act as a depot adjuvant and sustain the release of antigen to enhance immune activation as observed in rodent models of food allergy where matrix lipids are found to act as adjuvants for allergic responses to the allergen on injection (*e.g.*, Brazil nut). Alternatively, the lipids themselves may be directly immunostimulatory.

Lipids and other components of the food matrix have the potential to exert immunomodulatory effects. Lipids in particular have been exploited for their properties as oral adjuvants (immune stimulating complexes [ISCOMs], liposomes). Saponins act as adjuvants presumably through increased antigen uptake, but can also exert effects when administered at a different site than the antigen. Plant sterols and lectins can also be immunostimulatory. Although not a major allergen, peanut lectin has mitogenic activity and may therefore enhance systemic immune reactivity more generally. Additionally, the influence from food contaminants of microbial or fungal origin, such as lipopolysaccharides (LPS) or aflatoxin, respectively, may further complicate the immune response as they may act as adjuvants or modifiers of general immune status. An immunologic phenomenon thought to limit food allergy is oral tolerance, which can occur when ingesting soluble antigen leads to hyporesponsiveness to that specific antigen. Lipids have also been shown to influence oral tolerance and shape the character of the immune response to subsequent immunization in animal models. Furthermore, biophysical features of a matrix such as the pH can also influence oral tolerance induction, perhaps by modifying protein structure or solubility or by affecting digestion prior to or during immune system exposure [22].

The food matrix also affects protein modifications that may occur during processing. Maillard reactions between proteins and sugars can induce aggregates, which increases allergenicity by providing new IgE binding sites or potentiating more effective crosslinking of IgE molecules. For some allergens, glycation can form new epitopes for IgE binding, whereas the glycation of other allergens can reduce IgE binding. Other considerations include matrix-related thermostability and altered antigenicity due to protein interactions. For example, the antigenicity of ovomucoid is reduced when combined with wheat, perhaps due to interactions with wheat gliadins [23].

Finally, matrix issues confound measurement of proteins in foods, limiting understanding of exposure. The matrix in which an allergen is present might alter the reactivity in patient serum screening experiments, further complicating the determination of allergenic potential for the broader population.

### Danger signals from allergens: proteolytic action

What makes an allergen an allergen? This question has been the subject of much speculation and research, and was carefully considered and discussed at the 2012 symposium. The answer to this question is certainly not a simple characterization applicable to all proteins that have been identified as allergens. A first prerequisite for answering the question is to define what an allergen uniquely possesses. Two properties are part of the definition: 1) the capacity to trigger the immune system to start producing specific IgE antibodies, and 2) the capacity to elicit allergic symptoms via an IgE-mediated mechanism. Many allergens possess both properties, but some food allergens that are related to pollen allergens are allergens by virtue of their structural similarity to the original sensitizer from pollen; the food allergen itself lacks the capacity to induce IgE antibodies. The symposium discussion was limited to the capacity of an allergen to induce the IgE antibody induction pathway.

Probably the only prerequisite for the induction of IgE antibodies that holds true for all proteins is exposure. Without exposure, there can be no response of the immune system. The dose–response is, however, not a simple linear relationship, and other ‘danger signals’ favoring induction of IgE and the associated stimulation of Th2 cells come into play. A ‘danger signal’ that has attracted much attention is proteolytic activity of proteins. Most evidence for a role of proteolytic enzymatic activity in allergenicity comes from studies on cysteine proteases from house dust mites, *i.e.*, the group 1 allergens from *Dermatophagoides pteronyssinus* and *Dermatophagoides farinae*, Der p 1 and Der f 1, respectively. Three main targets of proteolytic activity have been described: the integrity of the physical barrier of epithelial cells, the innate immune response orchestrated by structural cells of this barrier (epithelial cells/keratinocytes), and the subsequent adaptive immune response.

In a series of elegant experiments reported around the turn of the century, Robinson and co-workers demonstrated that tight junctions are disrupted on exposure to Der p 1, thereby facilitating passage of protein across the epithelial barrier [24-26]. Disruption of the epithelial barrier in itself, however, does not definitively make a cysteine protease an allergen. It simply allows the mucosal immune system to be more easily exposed to any protein from the environment, in addition to the proteases themselves. Whether tight junctions of atopic individuals are more sensitive to proteolysis is not known.

Around the same time, Stewart and co-workers identified another consequence of the proteolytic activity of house dust mite proteases. On exposure to Der p 1 (and the serine protease Der p 9), cultured epithelial cells started producing the pro-inflammatory cytokines GM-CSF, IL-6 and IL-8, thus providing a cytokine milieu favoring induction of inflammatory adaptive immune responses [27]. Further work demonstrated that protease-activated receptor-2 (PAR-2) but not PAR-1 is at the basis of the activation of epithelial cells [28]. This was later confirmed by Jacquet and co-workers [29]. As for disruption of tight junctions, enzymatic activity may provide a favorable cytokine and chemokine milieu for induction of IgE and Th2 responses, but it does not provide an explanation for why one protein under these conditions induces an IgE response and another does not.

Additional studies on Der p 1 have shown the potential for multiple effects of a proteolytically active allergen. In 1995, Shakib and co-workers demonstrated that Der p 1 cleaves the low-affinity IgE-receptor on B cells (*i.e.*, CD23), thereby disrupting the negative feedback loop for IgE production [30,31]. They then showed with further work that Der p 1 also cleaves CD25, the α-subunit of the human T-cell IL-2 receptor [32]. The consequence of this is that proliferation of T cells and production of IFNγ is inhibited, thus favoring Th2 responses that would lead to B-cell support and IgE production. In addition to a direct effect on T cells, it was also demonstrated that dendritic cells (DCs) are affected by proteolytically active Der p 1 [33]. DCs produced less IL-12, and CD40 was cleaved, thus having an impact on activation of T cells with decreased IFNγ and increased IL-4 production. Several other potential pro-allergenic effects have been described, such a cleavage of DC-SIGN on DCs [34] and inhibition of indoleamine 2,3-dioxygenase production by DCs [35]. Overall, a combination of all of these effects on the adaptive arm of the immune system may indeed favor Th2 and IgE responses, although it should be stressed that these effects do not discriminate proteolytic enzymes from other proteins present in a pro-inflammatory milieu.

In summary, there is convincing evidence that cysteine protease activity provides opportunities for those proteins which are exposed to the mucosal barrier and immune system and subsequently induce a Th2-type immune response to act as sensitizing allergens. It does not, however, explain why some proteins, including proteases, exposed under these same conditions sometimes do induce a specific IgE response and sometimes do not induce the full IgE maturation process. The molecular level processes, in terms of how an enzyme that is also an allergen may cross the cellular barrier, is important to understand, regardless of whether the current models focus on primarily respiratory allergens. Certainly, lessons learned from allergens such as Der p 1 would have applicability for testing and characterizing the ability of any allergen to cross-over epithelial barriers which are necessary to induce sensitization. The link that is still missing most likely lies in the way antigen-presenting cells process proteins and subsequently present peptides to specific T cells. The question, “What makes an allergen an allergen?”, has not yet been answered, but proteolytic activity certainly contributes as a co-factor under certain conditions.

## Discussion

As noted in existing regulatory safety guidance and numerous publications, food allergy safety relies on an accumulation of characterization studies rather than a single test (i.e., a weight-of-evidence approach). Endogenous allergens in some food crops have also come under scrutiny with regard to potential increases due to the prevalence of GM food crops and, thus, new technology has been developed to support this aspect of addressing potential sensitization through exposure assessments. However, the challenge in identifying threshold levels or no-adverse-effect levels of most allergens [36], particularly in food crops such as soybean, limits the ability to single out GM foods from their non-GM counterparts. In fact, emerging research indicates a high degree of variability in the levels of endogenous allergens in plants and suggests that routine screening of GM food crops for endogenous allergen levels does not at this time provide utility for food safety. A similar form of variability can be observed in the way human populations respond to allergenic foods. Global surveys are far from comprehensive, but tend to show that exposure histories for individuals differ depending on their diets, an individual’s age at the time of exposure, and the inherent regional cultures from which they originate. This results in differences in prevalence for a given food allergen from one region to another (*e.g.*, North America versus Asia). As can be surmised, this variability limits simplistic categorizing of food allergens as having the same level of risk for individuals in all areas around the world.

Characterizing the biophysical aspects of protein allergens also remains an intense area of study because it is critical to understand the features that are unique to allergens compared to non-allergens. A long-term objective is to identify the common properties of allergens so that their potential to cause allergy can be predicted based on how they become exposed to the immune system, are processed by the immune system, remembered by the immune system, and elicit a clinically significant allergic response. One of the main factors in understanding food allergens is to recognize that they almost always interact with a complex food matrix before or during ingestion. These “matrices” are really the surrounding proteins, lipids, and carbohydrates that make up a plant seed or fruit, for example, and thereby influence how the human body interacts with allergens. At the cellular level, the observations of proteolytic effects on the immune system appear to be direct in some cases, but there is no direct cause and effect that is so unique as to place proteolytic allergens separately from allergens or to allow for prediction of a protein’s potential to sensitize. A biophysical feature such as possessing proteolytic enzyme activity contributes to allergenic potential for some proteins under some conditions and will remain an important consideration in the ongoing characterization of allergens.

Fully characterizing immune response to protein allergens continues to be a challenge, particularly in terms of defining the initial sensitizing response and using the information to predict elicitation and allergy prevalence. As more foods are distributed globally and as novel proteins are utilized in food crops, ensuring food allergy safety underlies the need for continued research to understand how protein allergens are different from other proteins. As it stands, regulatory guidance for new and novel foods/food proteins rests on characterizing the risk of elicitation [37,38]. There is no guidance that uses a measure of sensitization potential to predict allergy risk for proteins as a basis for risk determination, nor are there standardized safety approaches available. In fact, this has been made clear even through European regulatory-supported reviews of the science in this area [39].

## Competing interests

The authors declare that they have no competing interests.

## Authors’ contributions

SM, CB, MF-R, GSL, and RvR were speakers at the April 2012 Symposium on Sensitizing Properties of Proteins and contributed written summaries of their presentations to this manuscript. All authors read and approved the final manuscript.
